# Psycho-oncologists’ knowledge of cancer-related fatigue and the targets for improving education and training: results from a cross-sectional survey study

**DOI:** 10.1007/s00520-023-07882-5

**Published:** 2023-06-23

**Authors:** Marlena Milzer, Anna S. Wagner, Karen Steindorf, Senta Kiermeier, Martina E. Schmidt, Imad Maatouk

**Affiliations:** 1 Division of Physical Activity, Prevention and Cancer, German Cancer Research Center (DKFZ) and National Center for Tumor Diseases (NCT), Im Neuenheimer Feld 581, 69120 Heidelberg, Germany; 2 Medical Faculty, University of Heidelberg, Heidelberg, Germany; 3 Section of Psychosomatic Medicine, Psychotherapy and Psychooncology, Department of Internal Medicine II, Julius-Maximilian-University, Wuerzburg, Germany

**Keywords:** Cancer-related fatigue, Healthcare professionals, Psycho-oncology, Self-efficacy, Supportive care

## Abstract

**Purpose:**

To explore psycho-oncologists’ knowledge of cancer-related fatigue and their self-efficacy to intervene for fatigue. We further aimed to examine the role of fatigue in psycho-oncological training and derive specific suggestions for improvements.

**Methods:**

For this cross-sectional survey study, psycho-oncologists working in Germany were systematically recruited via an address directory or invited by training institutes or colleagues. The online survey encompassed questions on knowledge of fatigue guidelines and interventions, self-efficacy, counseling, and fatigue in professional training. Data were analyzed descriptively and using Mann-Whitney *U* tests. A logistic regression analysis was performed to identify variables linked to fatigue guideline knowledge.

**Results:**

Seventy two percent of the 144 surveyed psycho-oncologists stated not knowing any fatigue-specific guidelines. Those unaware of guidelines reported a lower self-efficacy to intervene for fatigue. However, despite low knowledge of the guidelines, more than 80% of the participants felt well informed about fatigue and reported high self-efficacy. Most participants were aware of the empirical evidence for psychotherapeutic interventions (95%); everyday physical activity, e.g., taking a walk (98%); yoga (82%); and mindfulness-based interventions (82%). Knowledge gaps existed concerning the evidence of resistance/endurance training for treating fatigue. Knowing that resistance/endurance training is an effective treatment was related to an increased frequency to recommend it to patients. Suggestions to improve training for psycho-oncologists included raising awareness earlier in the career path and offering multidisciplinary trainings for fatigue.

**Conclusion:**

To improve fatigue-related guideline knowledge among psycho-oncologists and enhance implementation into clinical practice multidisciplinary trainings are needed. Psycho-oncologists should play an important role in fatigue management.

**Trial registration:**

Clinicaltrials.gov, identifier: NCT04921644. Registered in June 2021.

**Supplementary Information:**

The online version contains supplementary material available at 10.1007/s00520-023-07882-5.

## Introduction

According to the National Comprehensive Cancer Network (NCCN) cancer-related fatigue (CRF) is a “distressing, persistent, subjective sense of physical, emotional, and/or cognitive tiredness or exhaustion related to cancer or cancer treatment that is not proportional to recent activity and interferes with usual functioning” [1]. Although diagnostic criteria were proposed by the Fatigue Coalition, CRF has not yet been included as a diagnostic entity in the International Classification of Diseases, presumably impeding proper diagnostics and management. The majority of cancer patients is affected by CRF during active treatment, and approximately one fourth of cancer survivors still experience moderate to severe fatigue symptoms months or years after treatment, impacting quality of life and daily functioning [2, 3]. Efforts have been made in the past years to promote research in the field of CRF, and clinical practice guidelines have subsequently been released by the NCCN, the European Society for Medical Oncology (ESMO), and the Canadian Association for Psychosocial Oncology (CAPO) [1, 4, 5]. Exercise training and psychosocial interventions like cognitive-behavioral therapy have been shown to be most effective in reducing CRF [1, 5, 6]. Mind-body interventions, e.g., yoga, and mindfulness-based stress reduction (MBSR) are also recommended for the treatment of CRF [1, 5]. Furthermore, a comprehensive practice of screening and information for CRF should be provided to all cancer patients [1, 5]. Despite these clear guideline recommendations, CRF is often disregarded and one of the most common unmet supportive care needs [2, 7, 8]. Indeed, several studies have already pointed to a knowledge-to-practice gap in various phases of CRF management including screening, information, counseling, and treatment [9–14]. Explanations for this knowledge-to-practice gap comprise knowledge deficits of CRF among healthcare professionals (HCP), a poor patient-provider-communication, and systemic barriers such as a lack of adequate reimbursement, time, and staff [11, 14]. Additionally, divergent views of patients and HCP concerning the relevance of CRF, with HCP underestimating prevalence and impact of CRF on patients’ daily life, might contribute to the current non-satisfactory situation [15].

According to the NCCN, multidisciplinary teams, including physicians, nurses, and psychologists, are most appropriate to ensure implementation of CRF guidelines in clinical practice [1]. Particularly experts in the field of psycho-oncology could play a crucial role in CRF care. However, to our knowledge, previous research has not yet investigated psycho-oncologists’ awareness of CRF. Therefore, the primary aim of our study was to assess psycho-oncologists’ knowledge of CRF guidelines and interventions and their self-efficacy to intervene for CRF. We further aimed to identify possible socio-demographic and professional variables linked to CRF guideline knowledge. Lastly, the current role of CRF in psycho-oncological education and training should be explored to derive targets for improvement.

## Methods

### Design and participants

This cross-sectional, observational study was part of the large-scale LIFT project (Clinicaltrials.gov, identifier: NCT04921644) investigating the current management of CRF in Germany. The online survey among psycho-oncologists was conducted between December 2021 and September 2022 and aimed to assess the psycho-oncologists’ perspective on CRF management. Individuals were eligible for participation if they worked as psycho-oncologists in Germany seeing at least one cancer patient per week for at least 1 year, had Internet access, and sufficient German language skills to understand the survey. The German Cancer Society (DKG) prescribes the following qualifications to be approved as a psycho-oncologist: masters’ (or comparable) degree in psychology, medicine or social pedagogy, training in psychotherapy, and advanced training in psycho-oncology certified by the DKG [16].

Throughout Germany, psychological and medical psychotherapists with psycho-oncological qualifications were randomly drawn from an address directory that is provided and kept up-to-date by the Cancer Information Service (CIS) [17]. The study team then invited the selected psycho-oncologists via postal mail to complete the survey. Reminder mails were sent approximately 3 weeks afterwards. To reach the targeted number of 140 cases, further psycho-oncologists were invited to study participation by colleagues or via mailing lists of a training institute for psycho-oncology. For survey completion, participants received an expense allowance of €15.

### Procedures and measures

After providing informed consent, participants completed the online survey, which took approximately 15 min. Items were self-developed, based on an Australian questionnaire [13] and had been pre-tested with psycho-oncologists. The survey included questions on sociodemographic and professional characteristics, perceived CRF-related knowledge, self-efficacy, knowledge of CRF interventions and guidelines, counseling on CRF and CRF in education, and training. Psycho-oncologists’ perceived CRF-related knowledge was assessed by the question “How well do you currently feel informed about CRF?” including the answer options “very poorly”, “rather poorly”, “rather well”, and “very well”. To assess self-efficacy to intervene for CRF agreement to the statement “I think that I can competently inform and counsel for CRF in my daily work” should be indicated on a four-point Likert scale. Knowledge of CRF guidelines was assessed by the yes-no question “Do you know (national or international) guidelines for CRF?”. Thereafter, knowledge of NCCN, ESMO, and CAPO guidelines and of guidelines somewhat addressing CRF, such as “Psycho-Oncology” [18] and “Palliative Care for patients with incurable cancer” [19] published within the German Guideline Program in Oncology, was rated on a four-point Likert scale with the scale endpoints “is not known to me” and “contents and recommendations of the guideline are well known to me”. To evaluate CRF guidelines, participants knowing any CRF guidelines were asked to indicate their agreement to five statements on a four-point Likert scale. To assess knowledge of interventions, participants had to decide for the listed interventions on a five-point Likert scale, ranging from “high evidence against” to “high evidence for”, if scientific evidence is available or if they are unable to judge it. Participants were further asked to provide information on counseling for CRF by indicating the frequency with which they recommend these interventions to patients. The extent to which CRF was covered in education and training was assessed via a Likert scale comprising the answer options “not at all/hardly”, “moderately”, and “comprehensively”.

### Statistical methods

Sample characteristics and answers to CRF-related questions are presented descriptively. Associations between sociodemographic/professional characteristics and CRF guideline knowledge were explored using a logistic regression model in which CRF guideline knowledge served as binary outcome variable. Odds ratios (ORs) and 95% confidence intervals (CIs) are reported. Variables considered as potentially relevant, based on theoretical considerations or previously performed univariate analyses, were simultaneously included in the model, i.e., age, gender, and leading position. Additionally, Mann-Whitney *U* tests were performed to clarify relationships between subgroups. For Mann-Whitney *U* tests, Pearson’s *r* was calculated, with |*r|* > .1 indicating small, |*r|* > .3 moderate, and |*r|* > .5 large effects. All statistical analyses were carried out using IBM SPSS version 29.0.0.0, with *p* ≤ .05 (two-tailed) considered statistically significant.

## Results

### Study population

Of the 162 psycho-oncologists who had provided informed consent, 18 were excluded due to missing data. Thus, the final sample consisted of 144 psycho-oncologists of which the majority (*n* = 123) was directly invited by study personnel to participate after having been systematically drawn from the address directory of the CIS (response rate: 34%). Additionally, 15 participants learned about the study by colleagues and the remaining six via mailing lists of the training institute for psycho-oncology.

Descriptive statistics of the study population are displayed in Table 1. The majority of our study population was female (83.3%), and the average age was 52 years (SD = 9.8). More than 60% had ≥ 10 years of working experience in oncology. Most of the psycho-oncologists worked in psychotherapy practices (64.6%) without leading position (74.3%). The majority had a license to psychotherapy practice (86.8%) and completed advanced training for psycho-oncology (97.2%).

**Table 1:** Sociodemographic and professional characteristics of the sample

	Total (*N* = 144)	
Variable	*M* or *n*	*SD* or %
Age [years]	51.6	9.8
< 40 years	21	14.6%
40–49 years	37	25.7%
50–59 years	52	36.1%
≥ 60 years	34	23.6%
Gender		
Female	120	83.3%
Male	23	16.0%
Diverse	1	0.7%
Working experience in oncology		
< 10 years	54	37.5%
10–20 years	68	47.2%
> 20 years	22	15.3%
Employment status		
Employed	57	39.6%
Self-employed	87	60.4%
Leading position		
Yes	37	25.7%
No	107	74.3%
Workplace		
Psychotherapy practice	93	64.6%
Certified cancer centers	20	13.9%
Oncological rehabilitation facility	19	13.2%
Other^a^	12	8.3%
Cancer patients per week		
1–5	69	47.9%
6–10	33	22.9%
11–20	26	18.1%
> 20	16	11.1%
License to psychotherapy practice		
Yes	125	86.8%
No	19	13.2%
Advanced training for psycho-oncology		
Yes	140	97.2%
No	4	2.8%

*M*, mean; *n*, number; *SD*, standard deviation

^a^Other: hospital with oncological focus (*n* = 4), other hospitals (*n* = 2), practice with oncological focus (*n* = 2), other practices (*n* = 1), cancer counseling center (*n* = 2), other (*n* = 1)

### Knowledge of CRF-related guidelines

Seventy two percent stated not to know any national or international guidelines for CRF. When probing more explicitly for specific CRF guidelines, 69%, 74%, and 84% indicated not to be aware of the NCCN, ESMO, and CAPO guidelines, respectively (Online Resource 1). Contents and recommendations of the NCCN guidelines were well known to 4% of the study population. This applied to 1% of the participants regarding the ESMO or CAPO guidelines. Seven percent were not aware of the psycho-oncology guidelines, whereas contents and recommendations of these guidelines were well known to 31%. Twenty seven percent did not know the guidelines for palliative care, whereas 46% indicated to know the contents partly or well.

**Fig. 1:**
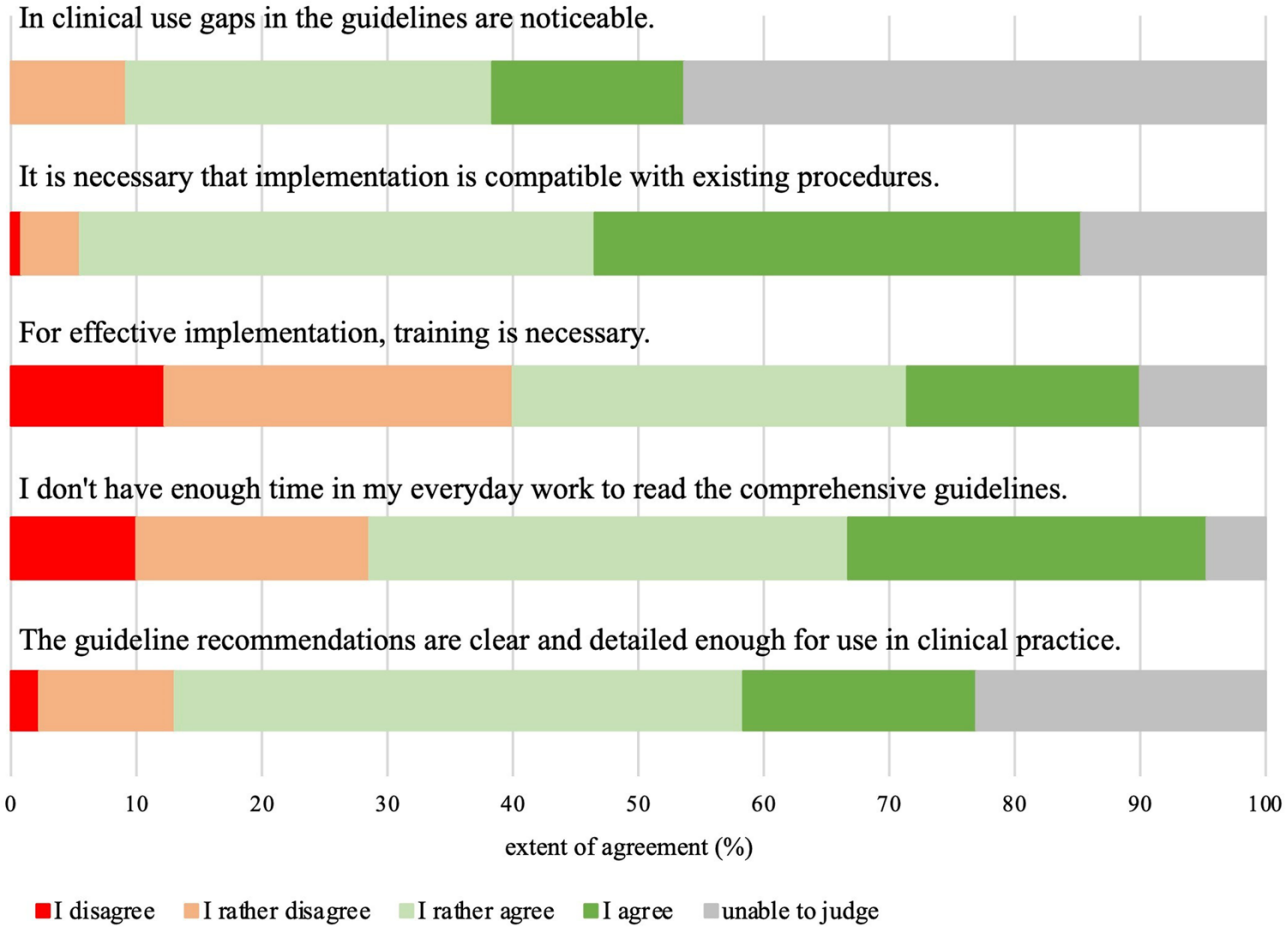
Psycho-oncologists’ views concerning clinical practice guidelines for fatigue

Psycho-oncologists’ views regarding the CRF guidelines can be found in Fig. 1. Although 64% of the psycho-oncologists knowing any CRF guidelines believed that the guideline recommendations are clear and detailed enough for use in clinical practice, 45% had noticed gaps. A lack of time prevents 67% from reading the guidelines. About 80% thought that implementation of guidelines must be compatible with existing procedures, and 50% stated that training is necessary for implementation.

The logistic regression analysis revealed a significant association between leading position and guideline knowledge, with psycho-oncologists in leading positions being more likely to know guidelines (OR = 2.88, CI [1.23; 6.74], *p* = .015). Female gender (OR = 0.20, CI [0.08; 0.54], *p* = .001) decreased the likelihood of reporting knowledge of CRF guidelines, while higher age increased the likelihood (OR = 1.05, CI [1.01; 1.10], *p* = .024). Adding further predictors like working experience did not significantly improve the model.

### Perceived CRF-related knowledge and self-efficacy to intervene for CRF

A majority of 73% felt rather well informed about CRF, while 11% stated to feel very well informed (Online Resource 2). Sixteen percent felt rather poorly informed about CRF. Regarding self-efficacy, almost 88% thought that they could competently inform and counsel for CRF in their daily work.

To enable the following analysis, three individuals who indicated “unable to judge” in the self-efficacy item were excluded. Significantly higher levels of self-efficacy were found in psycho-oncologists who knew CRF-specific guidelines when compared to those who did not (*M*_Rank_ = 87.96 vs. *M*_Rank_ = 64.05; *U* = 1354.50, *Z* = − 3.594, *p* < .001, *r* = − .303). Likewise, perceived CRF-related knowledge was greater in psycho-oncologists who knew guidelines than in the subgroup who did not (*M*_Rank_ = 83.12 vs. *M*_Rank_ = 68.27; *U* = 1676.00, *Z* = − 2.475, *p* = .013, *r* = − .206).

### Knowledge of CRF interventions

Psycho-oncologists’ knowledge of the scientific evidence of CRF interventions is depicted in Table 2. Fifty eight percent rated the evidence for psychotherapeutic interventions as high and 37% as rather high. Almost all were aware of the evidence for everyday physical activity, with 75% estimating the evidence as high and 23% as rather high. Regarding exercise training, 40% rated the evidence as high and 33% as rather high. Almost 13% reckoned that scientific evidence is against exercise training. Concerning yoga, 37% estimated the evidence as high and 45% as rather high. Eighty-two percent stated to know that mindfulness-based interventions are (rather) effective in treating CRF. Moreover, 79% indicated that the evidence for relaxation is (rather) high. Regarding the evidence of medication, nutrition-based interventions and mistletoe therapy views of psycho-oncologists were heterogeneous.

**Table 2:** Psycho-oncologists’ knowledge of interventions for cancer-related fatigue

	High evidence against recommending		Rather evidence against recommending		Unclear evidence		Rather evidence for recommending		High evidence for recommending		Unable to judge	
Intervention	*n*	%	*n*	%	*n*	%	*n*	%	*n*	*%*	*n*	*%*
Psychotherapeutic interventions (e.g., behavioral therapy, psychoeducation)	0	0.0	0	0.0	3	2.1	53	36.8	84	58.3	4	2.8
Physical activity in everyday life (e.g., taking a walk)	0	0.0	0	0.0	2	1.4	33	22.9	108	75.0	1	0.7
Exercise training (e.g., resistance/endurance training)	4	2.8	14	9.7	10	6.9	47	32.6	58	40.3	11	7.6
Yoga	0	0.0	1	0.7	16	11.1	65	45.1	53	36.8	9	6.3
Mindfulness-based interventions (e.g., qigong, MBSR^a^)	0	0.0	1	0.7	16	11.1	56	38.9	62	43.1	9	6.3
Relaxation (e.g., PMR^b^)	1	0.7	1	0.7	16	11.1	54	37.5	60	41.7	12	8.3
Medication	13	9.0	24	16.7	47	32.6	23	16.0	5	3.5	32	22.2
Nutrition-based interventions (e.g., nutritional counseling)	0	0.0	11	7.6	41	28.5	41	28.5	23	16.0	28	19.4
Mistletoe therapy	19	13.2	20	13.9	51	35.4	7	4.9	4	2.8	43	29.9

*n* = number; ^a^*MBSR*, mindfulness-based stress reduction; ^b^*PMR*, progressive muscle relaxation

### Counseling on CRF

Eighty four percent stated to often or almost always recommend psychotherapeutic interventions to patients showing signs of CRF (Online Resource 3). Almost all (98%) often or almost always recommended everyday physical activity, while 61% made the majority of their patients aware of exercise training. Twelve percent never, and 8% rarely recommended exercise training to patients affected by CRF. Nearly half of our study population often or almost always suggested their patients to do yoga and 62% to take up mindfulness-based interventions. Mann-Whitney *U* test revealed a significant difference in recommending exercise training to patients with CRF between psycho-oncologists who are aware vs. unaware of the empirical evidence for exercise training (*M*_Rank_ = 87.66 vs. *M*_Rank_ = 25.65 *U* = 246.00, *Z* = − 8.283, *p* < .001, *r* = − .695).

### CRF in education and training

**Fig. 2:**
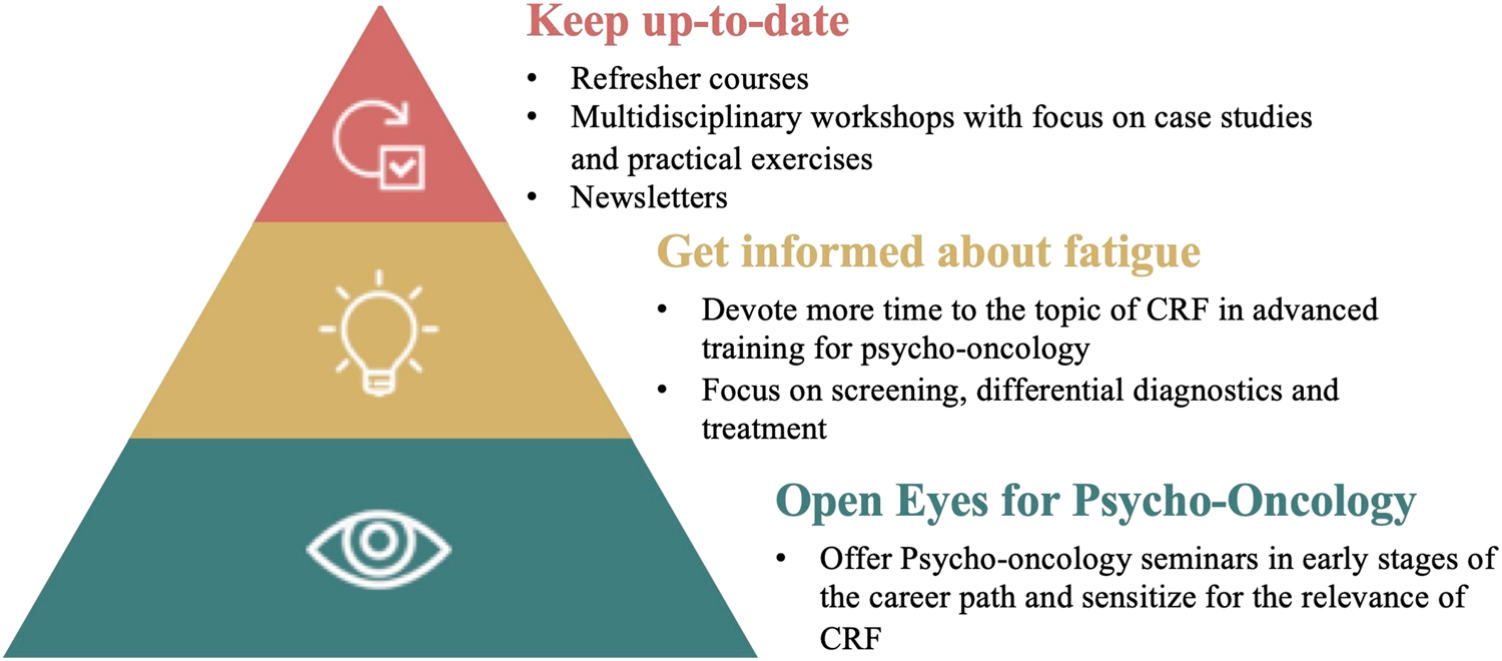
Psycho-oncologists’ suggestions for improving training for fatigue

More than 80% indicated that CRF is either not or hardly dealt with at university and during psychotherapy training. However, CRF is covered moderately (55%) or even comprehensively (39%) during advanced psycho-oncological training. Psycho-oncologists’ suggestions to improve education and training for CRF are presented in Fig. 2. First of all, they emphasized the importance of opening eyes for psycho-oncology among psychologists and related HCPs. According to the participants, this can be achieved by including the topic of psycho-oncology in early stages of the career path, e.g., within mandatory seminars in the psychotherapy training curriculum. Our participants believed that this might inspire psychologists to enter the field of psycho-oncology and raise awareness of relevant issues like CRF. Additionally, to expand knowledge of CRF among psycho-oncologists, our participants suggested to devote more time and resources to the topic of CRF in advanced psycho-oncology trainings. More specifically, as stated by our participants, it might be useful to offer all-day seminars on CRF for all future psycho-oncologists and provide the possibility of in-depth seminars for the particularly interested. Such fatigue seminars should primarily focus on practical aspects like screening, differential diagnostics, e.g., distinction from depression, and treatment of CRF. Finally, necessary steps to keep psycho-oncologists up-to-date regarding CRF should be considered. Participants recommended refresher courses and regular newsletters highlighting selected topics on the current state of research on CRF. Furthermore, multidisciplinary workshops concentrating on practical exercises were proposed with the aim of promoting exchange between different professional groups.

## Discussion

In this study, we investigated psycho-oncologists’ knowledge and self-efficacy to intervene for CRF. Furthermore, we assessed the role of CRF in education and training in order to identify concrete steps to improve CRF knowledge among psycho-oncologists.

Overall, we revealed knowledge gaps regarding CRF among psycho-oncologists. The majority was not aware of CRF-specific guidelines, and recommendations of the NCCN and ESMO guidelines for CRF were well known to only 4% and 1%, respectively. Similar to our findings, a Canadian study demonstrated HCPs’ low familiarity with CRF-specific guidelines [11]. In our study, CRF guideline knowledge was associated with higher age, male gender, and being in a leading position. We could not find any explanation for the unexpected gender effect, even when further scrutinizing potential confounders or collinearities. Since there were no significant gender effects regarding knowledge of CRF interventions, one might carefully hypothesize that men tended to overstate, while women tended to understate their guideline knowledge. Psycho-oncologists in leading positions might be more aware of the relevance of guidelines for quality assurance and have better access to trainings which are, according to our participants, required for successful implementation of CRF guidelines in clinical practice. Moreover, half of the study population noticed gaps in CRF guidelines. Thus, adaptations of CRF guidelines might be useful to promote and accelerate their application in clinical practice. As indicated by our participants and in accordance with previous research, it is important to provide concrete suggestions on how to ensure compatibility with existing practices [20].

Furthermore, a lack of knowledge emerged concerning the evidence of exercise training (resistance/endurance training) for treating CRF, with 7% stating that the evidence is unclear and 13% even assuming the evidence to be against exercise training. This resulted in a reluctance to recommend this measure to patients with CRF. Since exercise training is one of the most convincing interventions for CRF, this finding is alarming [6]. However, previous research yielded similar results suggesting that HCP are less likely to know and recommend interventions outside their expertise and discipline [11, 13]. Psycho-oncologists might further be concerned about overwhelming patients when recommending exercise training. It is, indeed, important to tailor counseling to the individual needs and conditions of the patient.

Numerous previous studies have also reported substantial CRF-related knowledge deficits in HCPs. For example, an Australian research group found that 28% of the surveyed HCPs were not able to mention any effective treatment for CRF [13]. A considerable number of participants in this study even recommended strategies like rest and sleep which lack empirical evidence, reinforcing the results of a former study [13, 21].

However, most psycho-oncologists in our study were aware of the empirical evidence for everyday physical activity, psychosocial interventions, mindfulness-based interventions, and yoga. Given that more psycho-oncologists thought that the evidence for yoga is rather high, but not very high and that not even half of the participants often/almost always recommended yoga to patients, show, however, that there is still room for improvement. This finding corroborates results of a previous study in which mind-body interventions were rarely recommended [14]. Yet, partially in contrast to the just discussed knowledge gaps, but consistent with a study conducted in the UK in which most HCPs felt confident in managing symptoms like CRF in cancer survivors [12], psycho-oncologists’ perceived CRF-related knowledge and self-efficacy to intervene for CRF were high in our study population. This was particularly true for those who knew CRF-specific guidelines.

HCPs’ knowledge deficits, together with gaps in the patient-provider-communication and systemic barriers such as a lack of time and reimbursement, presumably contribute to the knowledge-to-practice gap in CRF management [11, 14]. Therefore, increasing CRF-related knowledge among HCPs within specific trainings is one essential step towards a satisfactory CRF management. According to our participants, it is only in advanced psycho-oncological training that professionals learn about CRF, while the topic is hardly discussed at university and in psychotherapy training. Even though, for quality assurance, completion of a certified, advanced psycho-oncological training is increasingly required [18], a significant number of professionals without specific psycho-oncological qualifications work in psycho-oncological care. Therefore, it is important to sensitize for the relevance of CRF and to inform about this topic earlier in the career path. A previous study demonstrated that a training including education about CRF guidelines and teaching of patient-provider-communication skills and interactive elements could enhance guideline knowledge, self-efficacy and intent to adopt guidelines in clinical practice [22]. Along with the need for more comprehensive CRF workshops in advanced psycho-oncological training, our participants emphasized the importance of multidisciplinary trainings to promote exchange among different HCP groups. This is in line with guideline recommendations pointing out that multidisciplinary teams are most appropriate to ensure implementation in clinical practice [1]. Thus, investigating current multidisciplinary collaboration in CRF management with the aim of defining clear responsibilities will be subject to future analyses of our LIFT project.

### Study limitations

Despite systematic recruitment procedures and a response rate of 34%, a selection bias cannot be excluded. However, it can be assumed that particularly those who are highly motivated and interested in CRF participated in the survey. Actual knowledge gaps in the population of psycho-oncologists might thus be even more pronounced than the knowledge gaps identified in this study. Additionally, low numbers of cases in subgroups did not allow for more comprehensive analyses. Thus, our analyses should be considered explorative, and further possible determinants of CRF-related knowledge should be examined.

### Clinical implications

A lack of CRF guideline knowledge associated with lower CRF-related self-efficacy gives rise to the importance of thorough CRF-specific trainings. This is also illustrated by the fact that a significant number of psycho-oncologists in our study were not aware of the scientific evidence for exercise training or even suspected detrimental effects. Therefore, professional trainings early in the career path, e.g., during psychotherapy training, should be offered to raise awareness for the relevance of CRF. Further trainings should take place in a multidisciplinary setting, focus on the provision of up-to-date information on CRF, and include practical elements. Our study showed that psycho-oncologists rarely recommend interventions outside their discipline, e.g., exercise training, making clear that regular exchange between different professions, not only in trainings but also in daily clinical practice, is crucial. These results underline NCCN guideline recommendations saying that multidisciplinary teams are best suited to achieve progress in CRF management [1]. Multidisciplinary teams might facilitate the diagnostic process of CRF and ensure effective treatment. Since psycho-oncologists feel well informed about CRF and feel confident in treating patients affected by CRF, they should be comprehensively involved in CRF care.

## Conclusion

A majority of psycho-oncologists are not aware of CRF-specific guidelines. Furthermore, due to knowledge gaps concerning exercise training, a considerable number of patients have presumably not received this highly effective treatment option. Consequently, to promote implementation of CRF guidelines by improving HCPs’ knowledge, it is important to provide up-to-date information within multidisciplinary trainings. Further research into barriers of CRF management and strategies to resolve them is warranted.

## Supplementary information

ESM 1 (PDF 126 kb)

ESM 2 (PDF 107 kb)

ESM 3 (PDF 79.9 kb)

## Data Availability

Data can be made available to scientific cooperation partners upon reasonable request.

## Code availability

N/A
