# Supplementary material for: Psycho-oncologists’ knowledge of cancer-related fatigue and the targets for improving education and training: results from a cross-sectional survey study
Source: Support Care Cancer. 2023 Jun 23;31(7):412. doi: 10.1007/s00520-023-07882-5 (PMC10289967; doi:10.1007/s00520-023-07882-5)
Supplement: Supplementary file 1 — (PDF 126 kb) [file 520_2023_7882_MOESM1_ESM.pdf]

**Supplement 1**

*Psycho-oncologists' Awareness of Guidelines concerning CRF*

| | Is not known to me. | | I know it exists, but the contents are not familiar to me. | | I have read it once, so I know some of the contents. | | Contents and recommend-dations are well known to me. | |
|---|---|---|---|---|---|---|---|---|
| | *n* | % | *n* | % | *n* | % | *n* | % |
| NCCN Guideline | 99 | 68.8 | 28 | 19.4 | 12 | 8.3 | 5 | 3.5 |
| ESMO Guideline | 106 | 73.6 | 29 | 20.1 | 7 | 4.9 | 2 | 1.4 |
| CAPO Guideline | 121 | 84.0 | 21 | 14.6 | 1 | 0.7 | 1 | 0.7 |
| Psycho-Oncology[a] | 10 | 6.9 | 17 | 11.8 | 72 | 50.0 | 45 | 31.3 |
| Palliative Care[a] | 39 | 27.1 | 39 | 27.1 | 51 | 35.4 | 15 | 10.4 |

*Note.* [a]Guidelines published within the German Guideline Program in Oncology ("S3-Leitlinien").

**Article title:** Psycho-oncologists' knowledge of cancer-related fatigue and the targets for improving education and training: results from a cross-sectional survey study

**Journal name:** Supportive Care in Cancer

**Author names:** Marlena Milzer, Anna S. Wagner, Karen Steindorf, Senta Kiermeier, Martina Schmidt, Imad Maatouk

**Corresponding Author:**

Prof. Dr. Karen Steindorf

Division of Physical Activity, Prevention and Cancer (C110)

German Cancer Research Center (DKFZ)

Im Neuenheimer Feld 581

69120 Heidelberg, Germany

Phone: +49 (0) 6221-42 2351

E-Mail: k.steindorf@dkfz-heidelberg.de
