# Supplementary material for: Psycho-oncologists’ knowledge of cancer-related fatigue and the targets for improving education and training: results from a cross-sectional survey study
Source: Support Care Cancer. 2023 Jun 23;31(7):412. doi: 10.1007/s00520-023-07882-5 (PMC10289967; doi:10.1007/s00520-023-07882-5)
Supplement: Supplementary file 2 — (PDF 107 kb) [file 520_2023_7882_MOESM2_ESM.pdf]

Supplement 2

Psycho-oncologists' perceived CRF-related knowledge and self-efficacy to intervene for CRF

			Very poorly		Rather poorly		Rather well		Very well	
Item			*n*	%	*n*	%	*n*	%	*n*	%
How well do you currently feel informed about cancer-related fatigue?			0	0.0	23	16.0	105	72.9	16	11.1
	I disagree		I rather disagree		I rather agree		I agree		I cannot judge	
	n	%	*n*	%	*n*	%	*n*	%	*n*	%
I think that I can competently inform and counsel for fatigue in my daily work.	1	0.7	14	9.7	82	56.9	44	30.6	3	2.1

Note. n = number of cases.

Article title: Psycho-oncologists' knowledge of cancer-related fatigue and the targets for improving education and training: results from a cross-sectional survey study

Journal name: Supportive Care in Cancer

Author names: Marlena Milzer, Anna S. Wagner, Karen Steindorf, Senta Kiermeier, Martina Schmidt, Imad Maatouk

Corresponding Author:

Prof. Dr. Karen Steindorf

Division of Physical Activity, Prevention and Cancer (C110)

German Cancer Research Center (DKFZ)

Im Neuenheimer Feld 581

69120 Heidelberg, Germany

Phone: +49 (0) 6221-42 2351

E-Mail: k.steindorf@dkfz-heidelberg.de
