# Supplementary material for: Psycho-oncologists’ knowledge of cancer-related fatigue and the targets for improving education and training: results from a cross-sectional survey study
Source: Support Care Cancer. 2023 Jun 23;31(7):412. doi: 10.1007/s00520-023-07882-5 (PMC10289967; doi:10.1007/s00520-023-07882-5)
Supplement: Supplementary file 3 — (PDF 79.9 kb) [file 520_2023_7882_MOESM3_ESM.pdf]

Supplement 3

Frequencies with which psycho-oncologists recommend high-evidence interventions for cancer-related fatigue

	Never (0% of patients)		Rarely (1-25% of patients)		Sometimes (26-50% of patients)		Often (51-75% of patients)		Mostly/ almost always (76-100% of patients)		Unable to judge	
Intervention	*n*	%	*n*	%	*n*	%	*n*	%	*n*	%	*n*	%
Psychotherapeutic interventions (e.g. behavioral therapy, psychoeducation…)	1	0.7	5	3.5	14	9.7	35	24.3	86	59.7	3	2.1
Physical activity in everyday life (e.g. Taking a walk)	0	0.0	0	0.0	3	2.1	33	22.9	108	75.0	0	0.0
Exercise training (e.g. resistance/endurance training)	17	11.8	12	8.3	25	17.4	27	18.8	61	42.4	2	1.4
Yoga	9	6.3	19	13.2	47	32.6	25	17.4	44	30.6	0	0.0
Mindfulness-based interventions (e.g. qigong, MBSR[a])	4	2.8	12	8.3	38	26.4	31	21.5	58	40.3	1	0.7

Note. n = Number; [a] Mindfulness-based stress reduction.

Article title: Psycho-oncologists' knowledge of cancer-related fatigue and the targets for improving education and training: results from a cross-sectional survey study

Journal name: Supportive Care in Cancer

Author names: Marlena Milzer, Anna S. Wagner, Karen Steindorf, Senta Kiermeier, Martina Schmidt, Imad Maatouk

Corresponding Author:

Prof. Dr. Karen Steindorf

Division of Physical Activity, Prevention and Cancer (C110)

German Cancer Research Center (DKFZ)

Im Neuenheimer Feld 581

69120 Heidelberg, Germany

Phone: +49 (0) 6221-42 2351

E-Mail: k.steindorf@dkfz-heidelberg.de
